# Nationwide Tracking of A‐Lineage Spread in Western Honey Bees Using Haplotype Sharing, Local Ancestry Mapping, and Migration Inference

**DOI:** 10.1002/ece3.74574

**Published:** 2026-10-30

**Authors:** Mulusew Fikere, Dylan K. Ryals, Andrew H. Paterson, Brock A. Harpur

**Affiliations:** ^1^ Department of Entomology Purdue University West Lafayette Indiana USA; ^2^ University of Georgia Athens Georgia USA

**Keywords:** directional migration, gene flow, haplotype ancestry, honey bee, RFMix, spatial genomics, TreeMix

## Abstract

Understanding the spatial dynamics of genetic ancestry is essential for elucidating historical and contemporary patterns of gene flow among populations. Honey bees in the United States have a 400‐year history of importation, management, and adaptation to diverse environments. Genetic diversity is a key factor for colony health and resilience. Notably, the most genetically diverse populations in the country originate from the invasive A‐lineage (Africanized or ‘Killer’ honey bees). In this study, we leveraged a nationwide community‐science platform to assemble an extensive genomic dataset of U.S. honey bee populations and used it to ask how this invasive lineage is spreading across the country and how its spread has impacted honey bee genetic diversity nationwide. Using RFMix, we assigned local ancestry across the genome to capture fine‐scale ancestry patterns and applied TreeMix to genome‐wide allele frequency data to infer directional gene flow among U.S. populations. Our analyses revealed patterns consistent with a strong northward migration, with major source populations in southern states such as Texas (TX), Arizona (AZ), and Florida (FL), and primary targets in northern states including Michigan (MI), New York (NY), and Vermont (VT). Covariance estimates indicated strong genetic connectivity, particularly from TX and AZ. Spatial visualization of migration edges highlighted key routes of A‐lineage expansion. Together, these results provide the first genome‐wide evidence for a nationwide spread of A‐lineage ancestry in the U.S., offering insights to guide breeding strategies and predict the future dynamics of honey bee genetic diversity.

## Graphical abstract

This study investigates the spatial spread and genetic impact of A‐lineage (Africanized) ancestry in U.S. honey bee populations using a large, community‐science genomic dataset. By applying RFMix and TreeMix, we reveal strong northward gene flow originating from southern states such as Texas, Arizona, and Florida into northern regions, with high migration intensity and distinct expansion routes. These findings provide genome‐wide evidence of ongoing A‐lineage expansion and offer important insights for honey bee breeding and management strategies.

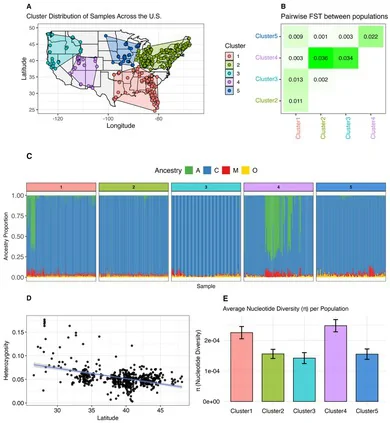

## 1 Introduction

Honey bees ( *Apis mellifera* ) are critical pollinators, playing an indispensable role in both natural ecosystems and agricultural productivity (Aslan et al. 2016; Harpur et al. 2012; Hung et al. 2018; Papa et al. 2022). In the United States (U.S.) alone, the economic value of honey bee‐mediated pollination is estimated as 15 billion dollars annually, supporting the yield and quality of over 90 commercial crops including almonds, apples, berries, and melons (Aukema et al. 2011; Khalifa et al. 2021; Losey and Vaughan 2006). However, honey bee populations in the U.S. and worldwide are under increasing pressure from diseases, parasites (e.g., *Varroa destructor* ), pesticide exposure, climate change, and habitat loss (Aurell et al. 2024; Nearman et al. 2025). Genetic diversity is a central determinant of resilience in both managed and wild bee populations, providing the raw material for adaptation and for selective breeding of traits such as disease resistance, climate tolerance, and productivity (Mattila and Seeley 2007; Ryals et al. 2024; Tarpy 2003).

The Western honey bee ( *Apis mellifera* L.) is a genetically diverse species with over 30 recognized subspecies grouped into at most seven distinct lineages divided by geography and genetics: The European M and C; Western Asian and Middle Eastern O and Y; and the African A, U, and L lineages (Dogantzis et al. 2021). In the U.S., honey bee populations are highly admixed due to multiple global introductions, primarily from European (M and C) sources (Carpenter and Harpur 2021). Historical evidence suggests that European honey bees were introduced into parts of the Americas by Spanish colonists before their documented introduction into what is now the United States. Documentary evidence indicates that honey bees were transported to the Caribbean during the sixteenth century, while historical accounts suggest they were subsequently established in New Spain, although the precise timing of their introduction remains uncertain because of incomplete historical records (Brand 1988; Crane 1999). In the 1600s, European colonists brought M‐lineage bees to North America, where they remained the predominant managed lineage until the late 1800s, when mass importations of C‐lineage bees occurred due to beekeepers' preference for phenotypes expressed by this group, including high honey production, rapid population growth, and low defensiveness (Carpenter and Harpur 2021). Today, most of the ancestry found in North American honey bees can be traced to C‐lineage sources (Guzman‐Novoa et al. 2024; Harpur et al. 2015). Given this history, we expect to observe widespread and high levels of C‐lineage ancestry across U.S. honey bee populations. In recent history, the United States saw the introduction and establishment of a new honey bee lineage. *Apis mellifera scutellata* , an African‐derived A‐lineage bee commonly referred to as the “Africanized” or “Killer Bee,” was introduced into Brazil in 1956, spread through South and Central America, and arrived in the U.S. in the 1990's (Carpenter and Harpur 2021).

The current distribution of the A‐lineage in the US appears constrained by limited cold tolerance which restricts their persistence in northern climates; however, warming environmental conditions have raised concerns about further northward expansion (Harrison et al. 2006; Oleksa et al. 2021). Because of their distinct behavioral traits, including heightened defensiveness, increased swarming propensity, and rapid colony turnover, the presence of A‐lineage ancestry carries major implications for beekeeping, commercial breeding, and colony management strategies. As a result, State and Federal agencies maintain active surveillance programs to monitor A‐lineage introgression due to its economic, ecological, and public‐safety impacts. Despite more than three decades of A‐lineage presence in the U.S., our understanding of their nationwide migration pathways, long‐term persistence, and genomic integration remains incomplete outside of at least one exceptional local study (Calfee et al. 2020). Their arrival created a large‐scale natural experiment in honey bee hybridization, where African‐ and European‐derived lineages have mixed to varying degrees across the Americas. Recent genomic studies demonstrate that both selection and hybridization have shaped this invasion, enabling A‐lineage ancestry to spread and persist even in the face of ecological barriers and intensive management practices (Calfee et al. 2020; Nelson et al. 2017).

Understanding how A‐lineage ancestry is distributed across the United States and how it has spread over time is crucial for designing effective conservation and breeding strategies. In this study, we leverage one of the most comprehensive genomic datasets assembled to date, spanning a nationwide collection of honey bee colonies with dense genotype information, to trace the migration history and genomic distribution of A‐lineage ancestry. We employ two powerful population genetic tools tailored for this study: RFMix (Maples et al. 2013) for local ancestry inference and TreeMix (Pickrell and Pritchard 2012) to reconstruct population history and gene flow.

Ancestry inference tools are fundamental for characterizing population structure, genetic admixture, and local ancestry patterns in genomic studies. These approaches combine statistical and machine learning frameworks to estimate ancestral origins at both global and local genomic scales. Among them, RFMix (Maples et al. 2013) is widely used for local ancestry inference, employing a conditional random field (CRF) model alongside reference population data to assign ancestry to specific chromosomal segments in admixed individuals (Atkinson et al. 2021). By integrating haplotype information with probabilistic modeling, RFMix provides high‐resolution ancestry deconvolution, making it especially valuable for populations with complex admixture histories. Its robustness and computational efficiency have led to widespread application in population genetics, evolutionary biology, and medical genomics (Atkinson et al. 2021; Honorato‐Mauer et al. 2025; Oliveira et al. 2024; Uren et al. 2020).

Complementing local ancestry inference, TreeMix reconstructs population history by modeling genome‐wide allele frequency covariance to infer population splits and historical gene flow (Pickrell and Pritchard 2012). By incorporating genetic drift and migration, TreeMix allows visualization of population trees with migration edges, facilitating detection of directional gene flow and admixture events (Rick et al. 2017). In this study, TreeMix is applied to characterize potential migration events and the directional spread of A‐lineage ancestry across the United States.

By integrating local ancestry inference and gene flow modeling on a nationwide, high‐resolution genomic dataset, this study provides new insights into the evolutionary dynamics of honey bee diversity in the United States. These findings are not only critical for scientific understanding of honey bee population structure, but also for informing future breeding and conservation efforts that aim to preserve the adaptive potential of U.S. honey bees. We hypothesize that U.S. honey bee populations retain identifiable A‐lineage haplotypes that generate distinct patterns of local ancestry and genomic introgression. We further propose that southern populations with high proportions of A‐lineage ancestry act as primary sources of northward gene flow, thereby shaping the spatial structure of haplotype sharing and migration intensity across the country. Finally, we hypothesize that A‐lineage introgression resulting from historical admixture influences the persistence and distribution of these haplotypes within U.S. honey bee populations.

## 2 Materials and Methods

### 2.1 Sample Information and Metadata

A total of 2359 worker honey bee samples (diploid individuals) were collected across the United States to capture broad geographic and population diversity. Samples were obtained through collaborative efforts involving commercial and small‐scale beekeepers, government personnel, and the study authors. Honey bee workers were sampled from both managed colonies and feral populations to ensure representation of diverse genetic backgrounds and colony management systems.

To minimize sampling redundancy and relatedness, each sampled individual originated from a separate colony. Associated metadata were recorded for each sample when available, including geographic location (state and GPS coordinates or nearest locality), collection year, colony management status (managed or feral, Figure S1B), and source information. Additional metadata, including beekeeper identification, breeding stock information, and environmental descriptors, were available for subsets of samples. Because the dataset was assembled through a community‐science framework, sampling intensity varied among geographic regions, reflecting differences in beekeeper participation and colony availability. To reduce potential spatial bias, analyses were conducted using population genetic approaches that account for unequal sampling density, and interpretations were focused on broad‐scale regional genetic patterns rather than fine‐scale local inferences. Furthermore, the large geographic coverage and inclusion of both managed and feral colonies helped mitigate overrepresentation of individual breeding operations or localized populations. Commercial honey bee management in the United States frequently involves interstate transport of colonies and the movement of package bees and queens, particularly from southern breeding regions. Consequently, inferred patterns of genetic connectivity likely reflect a combination of natural dispersal and human‐mediated movement. Because detailed colony transportation histories were unavailable for most samples, the study does not attempt to explicitly distinguish natural migration from anthropogenic movement. Instead, results are interpreted as representing realized gene flow within contemporary U.S. honey bee populations under current apicultural practices.

### 2.2 Genomic Data and Quality Control

Genomic data from all 2359 honey bee samples (Project: ID: PRJEB123676 and accession number ERZ29807913) were used for analysis (Figure S1A). DNA was extracted from thorax tissue using a lysis buffer containing proteinase K, followed by incubation at 65°C for 1 h and bead‐based purification. Library preparation was carried out by Gencove Inc. (Lincoln, NE, USA) using the Illumina Nextera DNA Flex kit. Libraries were quantified via fluorometry, pooled, and quality‐checked with an Agilent TapeStation. Sequencing was performed at ~5× coverage using an Illumina NovaSeq 6000, and reads were aligned to the Amel_HAv3.1 reference genome (Wallberg et al. 2019).

Genotype imputation was performed using Gencove's pipeline, incorporating ~1000 publicly available honey bee genomes following previous work in honey bees (see (Shultz et al. 2025 and Shultz et al. 2026)). The pipeline was based on an adapted version of (Rubinacci et al. 2023) and used Amel_HAv3.1 reference panel (Wallberg et al. 2019) for improved accuracy (Li and Stephens 2003). The imputed dataset included posterior genotype probabilities, from which 3.6 million high‐confidence biallelic SNPs were initially retained. To validate imputation, 5000 SNPs were randomly masked and imputed with BEAGLE v5.4 (Browning and Browning 2007), and accuracy was assessed using Pearson correlation. SNPs with < 99% posterior probability, more than two alleles, or minor allele frequency < 0.05 were removed, resulting in a final dataset of 593,756 SNPs across 2359 samples.

#### 2.2.1 Study Design and Analysis Workflow

To investigate the distribution and spread of A‐lineage ancestry in U.S. honey bee populations, we implemented an integrative genomic analysis pipeline combining local ancestry inference and gene flow modeling. For the local ancestry analysis, we utilized RFMix v2 (Maples et al. 2013). Two genotype datasets were prepared: a reference panel composed of 200 individuals with known ancestry and a target population dataset containing 2359 U.S. honey bee individuals representing managed and feral colonies across the United States.

The reference panel (*N* = 200) represented the major honey bee evolutionary lineages, including the African (A), Central European (M), Eastern European (C), and Middle Eastern/Western Asian (O) ancestries (ACMO). Reference individuals were obtained from globally representative populations previously characterized in Dogantzis et al. (2021) and Carpenter and Harpur (2021). These populations were selected based on documented subspecies origins, geographic representation, and prior population genetic validation, and are widely considered representative reference populations for their respective evolutionary lineages. Both reference and target datasets were phased independently to generate haplotype‐resolved data, which were then used as input for RFMix. The analysis produced SNP‐level haplotype ancestry assignments and genome‐wide ancestry proportions, enabling the assessment of the genomic extent and distribution of A‐lineage introgression across U.S. honey bee populations. For population‐level historical analysis, we employed TreeMix (Pickrell and Pritchard 2012). The phased target dataset was converted into PLINK binary format (Chang et al. 2015), and allele frequencies were estimated using PLINK based on predefined geographic/regional groups. This step generated SNP‐level allele frequency data stratified by population. The quality‐controlled SNP dataset was used without additional LD pruning to retain genome‐wide marker density, while linkage disequilibrium was accounted for in TreeMix. TreeMix was run using windows of 500 SNPs to account for linkage disequilibrium, with up to 20 migration events allowed as an exploratory upper bound. This value was not based on a specific biological expectation but was chosen to allow a broad range of potential migration scenarios. For the current analysis, the model allowing up to 20 migration events was used as the working model. TreeMix was fitted using five geographic clusters, and the inferred cluster‐level migration relationships were subsequently projected onto the U.S. states represented within each cluster for geographic visualization. The analysis generated a maximum‐likelihood tree of population relationships, migration edges indicating inferred gene flow among populations, and a covariance matrix of allele frequencies among populations.

### 2.3 Haplotype Sharing Among U.S. States Based on Ancestry Assignments From RFMix

The shared haplotype estimate represents a weighted measure of haplotype sharing across chromosomes, by accounting ancestry‐sharing proportions across all chromosomes to obtain a genome‐wide summary (Figure S3). After extracting genomic segments assigned to specific ancestries (“A” and “C”), we linked each haplotype to the state in which it was sampled and identified haplotypes present in individuals from more than one state to quantify shared ancestry. State boundaries were used as a geographic reference rather than as biologically meaningful population units. Shared haplotypes were defined using two complementary approaches: (1) identifying identical haplotypes (based on sample–haplotype identifiers) occurring in multiple states, and (2) calculating, for each chromosome, the proportion of genomic windows in which haplotypes from different states carried the same ancestry assignment, followed by averaging these proportions across chromosomes. Haplotypes sharing ≥ 20% of their ancestry segments were considered connected. This threshold was used as a visualization criterion to highlight meaningful haplotype sharing and reduce weak connections; it was not intended to represent a biological or statistical significance threshold. We then counted the number of shared haplotypes per state and categorized states according to their level of sharing, providing a chromosome‐informed genome‐wide framework to characterize regional patterns of gene flow.

### 2.4 Analysis of Genetic Diversity and Neutrality

Using the same set of SNPs as above, individuals were assigned to four reference populations (A, C, M, and O) based on sample identity. Genetic differentiation among populations was quantified using the fixation index (Fst) calculated for all pairwise comparisons (Weir and Cockerham 1984). Within each population, nucleotide diversity (π) (Nei and Li 1979) and Tajima's D (Tajima 1989) were estimated in non‐overlapping 10‐kb windows to evaluate within‐population variation and departures from neutrality.

To investigate spatial patterns of genetic diversity, we applied k‐means clustering (Ikotun et al. 2023) based on the great‐circle distance between the geographic coordinates (latitude and longitude) of all samples using the *distHaversine* function in the R package geosphere (Hijmans 2024). This analysis identified geographically coherent groups representing distinct regional groups. The optimal number of clusters (*k* = 5) was determined using the within‐cluster sum of squares (WCSS) and the elbow criterion (Ikotun et al. 2023). Each sample was subsequently assigned to one of five geographic/regional groups (Cluster1—Cluster5). SNP‐based clustering was not performed because the lineages show low genetic differentiation (low Fst), which would make genetic clustering noisy and difficult to interpret. Using geographic information provides clear, interpretable regional groupings relevant to our study. For each cluster, we calculated π and Tajima's D to assess within‐cluster diversity and neutrality and estimated pairwise Fst values to quantify genetic differentiation among geographic/regional groups. All analyses were conducted using VCFtools v1.6 (Danecek et al. 2011) and R packages (R Core Team 2024).

To further examine how geography influences genetic variation and connectivity among U.S. honey bee populations, we conducted two complementary analyses: a diversity cline test and an isolation‐by‐distance test. For the diversity cline, we calculated sample heterozygosity using VCFtools v1.6 and hierarchically clustered samples located within 10 km to account for spatial autocorrelation (Figure S5). We then applied a linear model to assess the relationship between mean heterozygosity in each cluster and the latitude of its centroid. For the isolation‐by‐distance analysis, we randomly selected one individual per cluster to minimize spatial bias and retained only SNPs with a minor allele frequency (MAF) > 1% and ≥ 90% completeness. After linkage disequilibrium pruning (*r* ^2^ < 0.2), 23,744 SNPs remained. Pairwise relatedness was estimated using the *make‐rel* command in PLINK v1.9, and geographic distances were computed as great‐circle distances using the *distHaversine* function from the geosphere R package (Hijmans 2024). The significance of the correlation between genetic and geographic distances was evaluated using a Mantel test with 1000 permutations implemented in the *mantel. test* function from the ape R package.

### 2.5 Regression Analysis of Ancestral Proportions and Haplotype Lengths

Haplotype‐specific ancestry tracts inferred by RFMix were used for downstream analyses. RFMix assigns local ancestry to each phased haplotype separately, producing ancestry labels along the genome for haplotype 1 and haplotype 2 of each individual. For each haplotype, contiguous genomic segments assigned to the same ancestry were identified and defined as ancestry tracts. These contiguous tracts were then aggregated across the genome and their physical lengths were calculated by summing base‐pair distances and converting them to megabases. For each tract, genomic midpoints were computed and subsequently merged with individual‐level metadata, including latitude, longitude, and other covariates, for regression analyses of ancestry proportions and tract length distributions. To assess geographic patterns, we analyzed the relationship between latitude and ancestral proportions (A, C, M, O) by averaging genome‐wide values across samples at similar genomic positions and fitting linear models (Ancestral proportion ~ Latitude). Similarly, linear regressions were performed to examine the association between ancestry haplotype length and latitude/longitude for each lineage. Samples were analyzed at the individual level, and apiary was not included as an explicit covariate or random effect in the regression models. Thus, individuals from the same apiary were treated as independent observations, with latitude and longitude representing the geographic predictors. Model fit was evaluated using *R* ^2^, *p*‐values, and 95% confidence intervals, with ANOVA testing the significance of latitude effects. Regression lines, confidence intervals, and regression equations were visualized to illustrate the patterns across lineages.

### 2.6 Gene Ontology Enrichment Analysis of Ancestry

To investigate the biological functions associated with genomic regions exhibiting elevated ancestry assignment probabilities, Gene Ontology (GO) enrichment analysis was performed on genes located within ancestry‐associated genomic regions. Candidate regions were identified from local ancestry inference as genomic intervals containing SNPs with ancestry assignment probabilities exceeding two standard deviations above the regional mean (2‐SD criterion). These ancestry‐associated regions were intersected with the *Apis mellifera* reference genome annotation (Amel_HAv3.1; NCBI Assembly GCF_003254395.2) using BEDTools (v2.31.0) to identify overlapping gene models. Gene identifiers were extracted from the corresponding GTF annotation file, yielding a non‐redundant set of candidate genes. GO enrichment analysis was conducted in R (v4.x) using the topGO Bioconductor package (Alexa and Rahnenführer 2023). A curated honey bee gene‐to‐GO annotation database was used to associate genes with Gene Ontology terms. Genes with GO annotations constituted the background gene universe, while genes overlapping the ancestry‐associated regions were treated as the candidate gene set. Enrichment was evaluated for the Biological Process ontology using the weight01 algorithm and Fisher's exact test, which accounts for the hierarchical structure of the Gene Ontology and reduces dependency among related GO terms. GO terms represented by fewer than ten annotated genes were excluded (nodeSize = 10) to reduce the influence of sparsely annotated categories.

### 2.7 TreeMix Analysis for Inferring Population Splits and Migration Events

Because RFMix does not infer directionality of migration, we also applied TreeMix to explore source–sink relationships among regions. We did so use whole‐genome SNP data. Genotype data were first processed in PLINK format (Chang et al. 2015), and allele frequencies were estimated for the population based on predefined groupings in a population map file. For each population, allele frequency $\left({\overset{`}{p}}_{i}\right)$ was estimated as the ratio of reference allele count $\left(x_{i}\right)$ to the total allele count $\left(n_{i}\right)$, where $\overset{`}{p_{i}}$ = $x_{i}$/$n_{i}$. The TreeMix algorithm (Pickrell and Pritchard 2012) assumes that allele frequency changes due to genetic drift follow a multivariate normal distribution and estimates a covariance matrix that reflects shared ancestry and migration events. The allele frequency drift is modeled as $∆p=\frac{1}{2N_{e}}p\left(1-p\right),$ where Ne is the effective population size and p is the allele frequency at a given locus. Residual fit analyses were used to assess model adequacy and to identify populations whose genetic relationships could not be fully explained by a tree alone, suggesting historical gene flow.

To determine the directionality of migration events, TreeMix analyzes the pattern of allele frequency covariance between populations. Migration edges are inferred in the direction that most effectively reduces the residuals between observed and expected covariances. The source and target of gene flow are determined by examining the orientation of the migration arrows on the graph, where the arrow typically points from the source (donor) to the recipient (target) population. Additionally, TreeMix reports the weight of each migration edge, which reflects the proportion of ancestry contributed by the source population. By integrating these statistical signals with the topology of the tree, TreeMix can distinguish between shared ancestry and post‐divergence gene flow of lineage across the United States.

### 2.8 Association Between Climate and Lineage Introgression

To evaluate whether climatic conditions were associated with patterns of lineage introgression, we obtained monthly and annual climate data for the period 2019–2024 across the contiguous United States from the National Oceanic and Atmospheric Administration (NOAA). Climate variables were extracted at each honey bee sampling location using the geographic coordinates of each colony at 10‐km spatial resolution. For the present analysis, we focused on annual minimum temperature (tmin) because low winter temperatures have been proposed as a major factor influencing the northward distribution of African‐derived honey bees (Southwick et al. 1990). Annual minimum temperature was calculated by averaging values across the 2019–2024 period for each sampling location. Global ancestry proportions for the A‐ and C‐lineages were estimated using RFMix, and the average haplotype length (Mb) for each lineage was calculated for every individual. Separate linear regression analyses were performed to evaluate the relationships between annual minimum temperature and (i) global ancestry proportion and (ii) average haplotype length for both the A‐ and C‐lineages.

## 3 Results

### 3.1 Genetic Diversity and Distribution of Ancestry Nationwide

To characterize nationwide patterns of honey bee diversity and ancestry, samples were assigned to five geographically coherent regional clusters identified by k‐means clustering of sampling locations (see Materials and Methods; Figure 1A). These geographically defined clusters were used as a framework for comparison rather than as discrete genetic populations. The PCA showed substantial genetic variation along the first two principal components (PC1 = 50.4%, PC2 = 13.6%; Figure S9), while pairwise Fst values indicated low genetic differentiation among geographic groups (*F*st = 0.001–0.036; Figure 2B). The greatest genetic differentiation was observed between the Mid‐Atlantic region (Cluster 2) and the Southwestern U.S. (Cluster 4), corresponding to regions that differ in climate, beekeeping practices, and the prevalence of A‐lineage haplotypes (Figure 1C). In contrast, the lowest differentiation occurred between the Mid‐Atlantic region (Cluster 2) and the Northern U.S. (Cluster 5). Honey bees across the country exhibited ancestries consistent with historical importation records, including A, M, C, and O lineages (Carpenter and Harpur 2021). We predicted that southerly populations would have increased A‐lineage ancestry due to the northward movement of this invasive population. Consistent with this expectation, only geographic/regional Group 1 and 4 (those in the South) showed elevated levels of A‐lineage ancestry (Figure 1 and Figure S2). We assessed whether the genetic differentiation observed among geographic/regional group was driven by differences in ancestry composition (A vs. C lineages) and found that most cluster comparisons showed very low genome‐wide differentiation, with elevated Fst values largely attributable to differences in ancestry composition rather than substantial divergence within the predominant C lineage (Figure S6).

**FIGURE 1:**
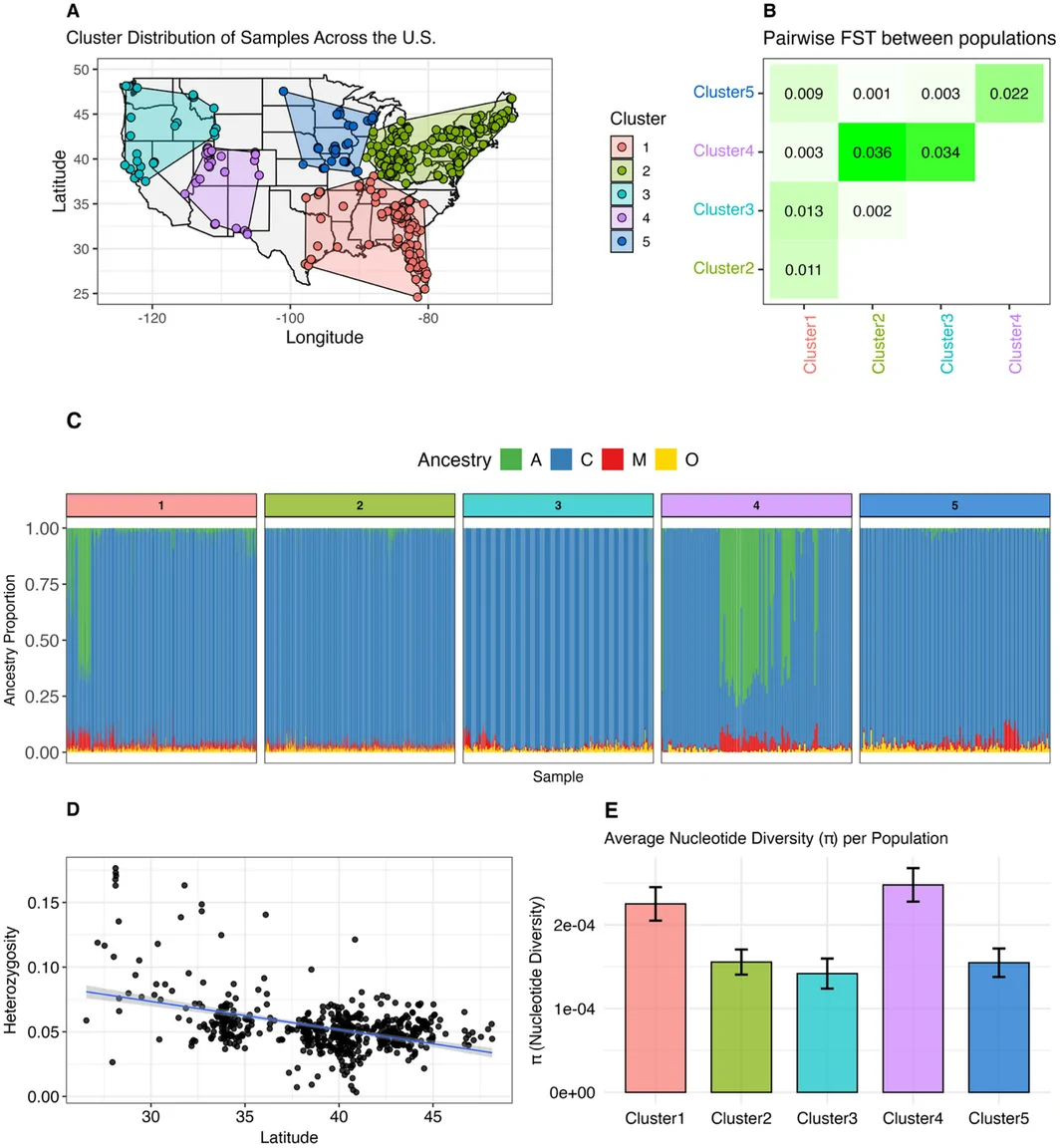
Genetic diversity and population differentiation using geographical regions (A), (B) pairwise *F*st values, and (C) Genome‐wide global ancestry proportions for 2359 individuals, grouped by ancestry categories (A, C, M, O) and sorted by latitude in ascending order. (D) Diversity cline: Significant negative relationship between latitude and heterozygosity, indicating a moderate decline in genetic diversity at higher latitudes among U.S. honey bee populations. (E) nucleotide diversity (π).

**FIGURE 2:**
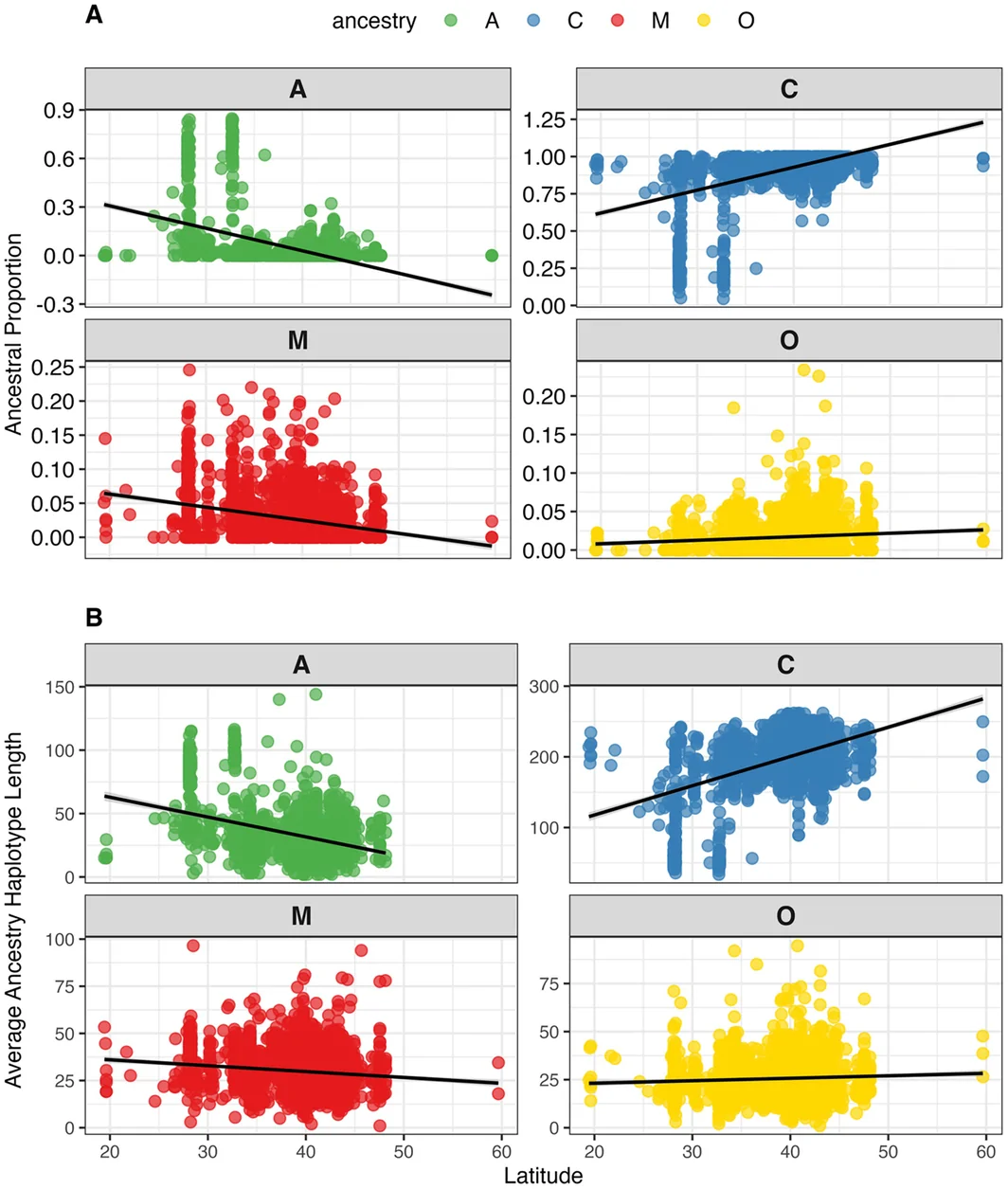
Genome‐wide ancestry patterns and spatial distribution in U.S. honey bee populations. (A) Regression analysis of ancestry proportion against latitude, showing spatial patterns of haplotype distribution. (B) Regression analysis of average ancestry haplotype length (Mbp) against latitude, showing spatial patterns of haplotype distribution.

### 3.2 A‐Lineage Is Restricted to the Southern US and Introgression Increases Genetic Diversity

The observation of elevated A‐lineage ancestry in southern populations was further investigated across all samples (Figure 1C and Figure S3). Regression of RFMix‐derived global ancestry proportions revealed distinct clinal patterns across the four major evolutionary lineages (A, C, M, and O) (Figure 2A and Figure S4). A‐lineage ancestry exhibited a moderate negative association with latitude (slope = −0.0139 per degree, *R* ^2^ = 0.223, *r* = −0.472, *f* ^2^ = 0.286, *p* < 2 × 10^−16^), indicating that African‐derived ancestry is concentrated in southern populations and declines toward the north. In contrast, C‐lineage ancestry displayed a moderate positive association with latitude (slope = 0.0154 per degree, *R* ^2^ = 0.224, *r* = 0.474, *f* ^2^ = 0.289, *p* < 2 × 10^−16^). M‐lineage ancestry showed a weaker negative association with latitude (slope = −0.00192, *R* ^2^ = 0.0761, *r* = −0.276, *f* ^2^ = 0.0823, *p* < 2 × 10^−16^), whereas O‐lineage ancestry showed a very weak positive association (slope = 0.000455, *R* ^2^ = 0.0106, *r* = 0.103, *f* ^2^ = 0.0107, *p* < 2 × 10^−16^) and remained relatively uniform across the sampled latitudinal range (Figure 2A). Haplotype tract length also varied geographically, with C‐lineage ancestry occurring in longer tracts toward northern populations and shorter tracts toward the south, whereas A‐lineage ancestry showed the opposite pattern (Figure 2B). In particular, mean physical A‐lineage tract length decreased significantly with increasing latitude (*β* = −0.092 Mb per degree, 95% CI = −0.102 to −0.082, *R* ^2^ = 0.182, *p* < 2.2 × 10^−16^), indicating progressively shorter A‐lineage ancestry tracts toward northern populations. We additionally find significant differences in the genetic diversity of samples with ancestry assignments from each lineage such that those with more A‐lineage ancestry are more genetically diverse than those with more C (Figure S7).

Finally, we found clinal variation in genetic diversity. Southerly geographic/regional groups (1 and 4) had higher overall genetic diversity than all other groups (Figure 1D). And this was directly related to differences in A versus C lineage presence (Figure 1E and Figure S5). We also find clinal variation in genetic diversity nationwide. More northern (C‐lineage) samples tended to have lower genetic diversity than more southern (A‐lineage) ones. We detected a significant negative relationship between latitude and heterozygosity (*r* ^2^ = 0.19; *F* = 134.8; df = 1, 559; *p* < 2.2 × 10^−16^), indicating a moderate decline in genetic diversity at higher latitudes among U.S. honey bees (Figure 1D). Populations at lower latitudes exhibited higher heterozygosity, whereas northern populations showed reduced genetic diversity. This latitudinal gradient suggests systematic geographic structuring of genome‐wide diversity across the United States.

### 3.3 Distribution of Global Ancestry Proportions Across the Genome

To assess the genome‐wide distribution of ancestral contributions, we summarized the RFMix global ancestry proportion estimates for 2359 individuals (Figure 3). The analysis focused on four major ancestry components: A, C, M, and O. The density curves reveal distinct patterns of ancestry representation, with ancestry group C exhibiting the highest and broadest distribution across individuals, followed by A‐lineage. A notable bimodal distribution of C‐lineage ancestry indicates that individuals tend to cluster toward either high or low C ancestry proportions, with relatively fewer individuals showing intermediate levels. This pattern is consistent with the presence of individuals with predominantly C ancestry alongside those with substantial A and M ancestry contributions. In contrast, the M and O ancestry components show more restricted and lower‐frequency distributions. These ancestries tend to cluster at low proportions (< 0.05), with few individuals displaying substantial contributions. The relatively narrow distributions of M and O suggest that these ancestry components contribute only modestly to most genomes in the dataset.

**FIGURE 3:**
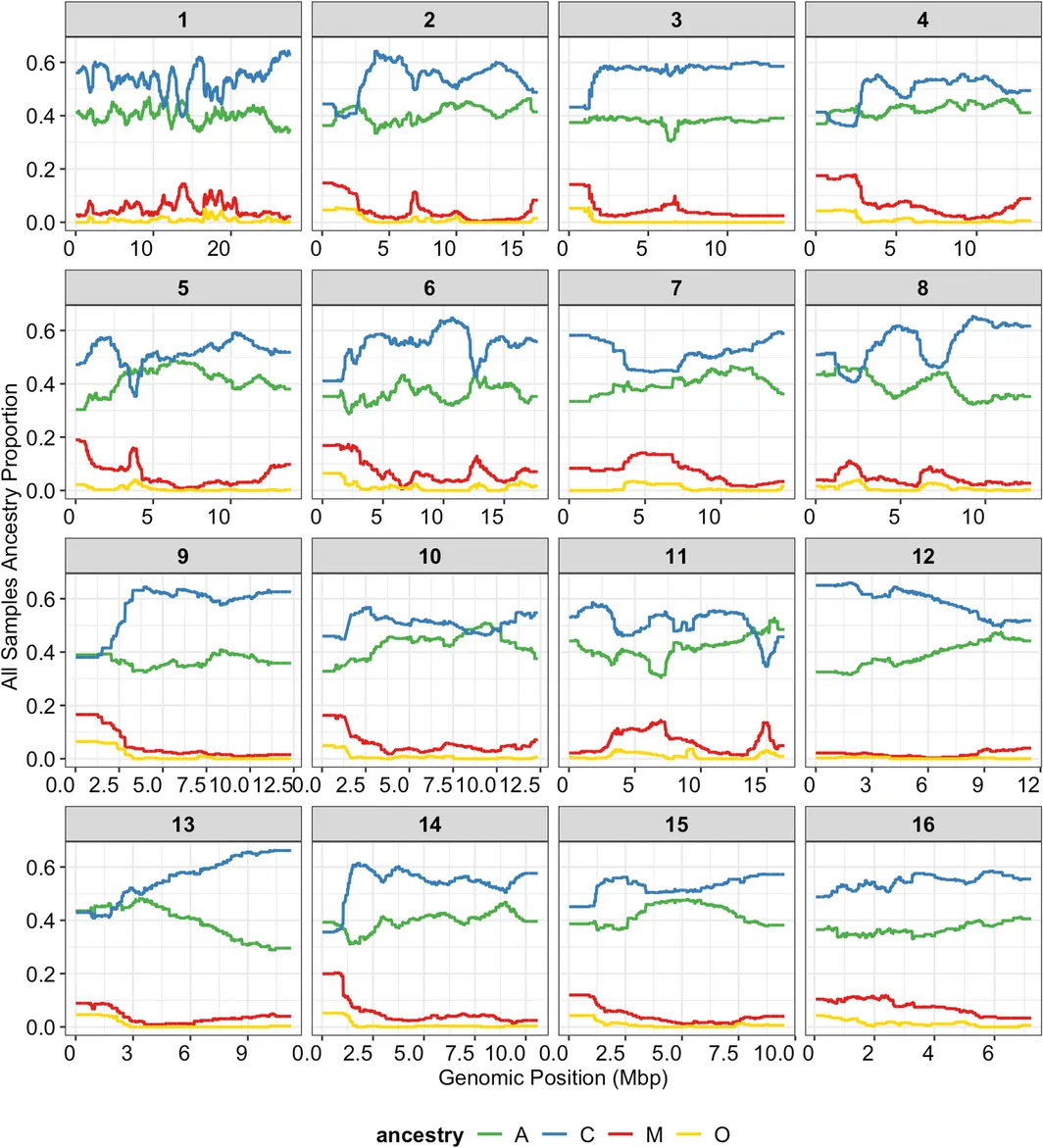
Genome‐wide global ancestry proportions for 2359 individuals across all chromosomes.

### 3.4 Gene Ontology Enrichment of Ancestry Candidate Genes

To gain insight into the biological functions represented within genomic regions exhibiting elevated ancestry assignments, we performed Gene Ontology (GO) enrichment analysis on genes overlapping ancestry‐associated genomic peaks (Figure 4 and Table S2). GO enrichment analysis revealed several lineage‐associated functional patterns among genes located within ancestry‐associated genomic regions (Table 1 and Table S2; Figure S7). Ancestry A‐associated regions contained 2027 candidate genes, of which 1886 were represented in the GO database. Several immune‐ and reproductive‐related processes showed nominal evidence of enrichment, including positive regulation of the immune system, innate immune response, male meiosis cytokinesis, and oocyte development; however, none remained significant after multiple‐testing correction (minimum FDR = 0.0744). In contrast, two developmental and morphogenetic processes, imaginal disc‐derived leg joint morphogenesis and embryonic hindgut morphogenesis, were significantly enriched among Ancestry C‐associated genes. Ancestry M showed significant enrichment for unsaturated fatty acid biosynthesis, indicating an overrepresentation of genes involved in lipid metabolic processes within M‐associated regions. Ancestry O exhibited the broadest enrichment profile, with significant associations spanning sensory perception, terpenoid and lipid biosynthesis, germ cell migration, and signal transduction. Notably, the strongest O‐lineage enrichment involved detection of chemical stimuli associated with sensory perception, while terpenoid and unsaturated fatty acid biosynthetic processes showed high gene ratios despite involving relatively small numbers of annotated genes (Table 1). These enrichment results provide functional context for the genomic regions associated with different ancestry components but should be interpreted as exploratory associations rather than direct evidence of selection or adaptive differentiation. Overall, the results suggest that ancestry‐associated regions encompass genes representing diverse developmental, metabolic, sensory, and signaling functions, with developmental and morphogenetic functions represented among C‐associated regions, lipid biosynthesis among M‐associated regions, and sensory, metabolic, and signaling functions among O‐associated regions.

**FIGURE 4:**
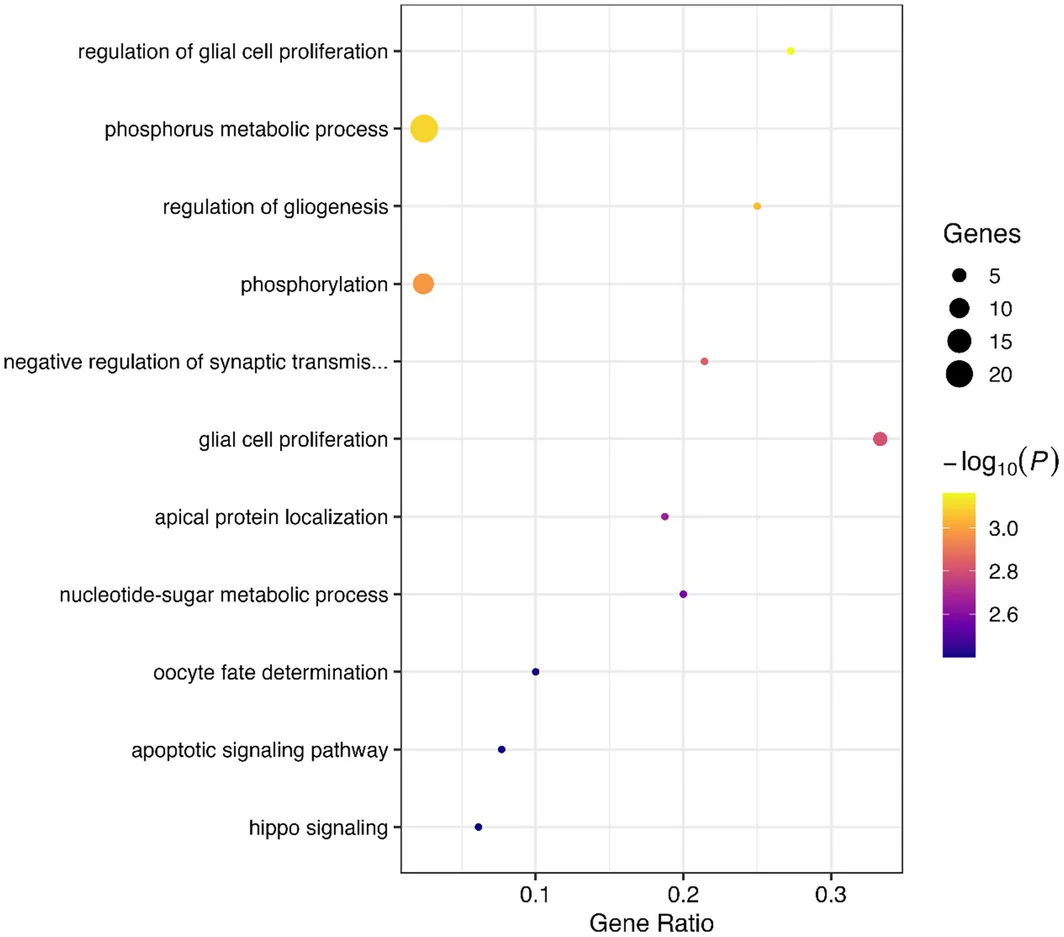
Functional characterization of genes within Ancestry A‐associated genomic regions. Dot plot showing the top‐ranked Gene Ontology (GO) Biological Process terms identified using topGO. Dot size represents the number of candidate genes associated with each GO term, color indicates nominal significance (−log_10_ *p*), and the x‐axis represents the gene ratio. Terms are ranked by nominal Fisher's exact test *p*‐value.

**TABLE 1:** Gene Ontology biological process terms associated with ancestry‐associated genomic regions.

Ancestry	Candidate genes	GO‐matched genes	GO term	Annotated	Significant	Gene ratio	*p*	FDR
A	196	141	Regulation of glial cell proliferation	11	3	0.273	6.9 × 10^−4^	0.0260
A	196	141	Phosphorus metabolic process	860	21	0.024	8.1 × 10^−4^	0.0260
A	196	141	Regulation of gliogenesis	12	3	0.250	9.0 × 10^−4^	0.0260
A	196	141	Phosphorylation	460	11	0.024	1.06 × 10^−3^	0.0260
A	196	141	Negative regulation of synaptic transmission	14	3	0.214	1.46 × 10^−3^	0.0260
A	196	141	Glial cell proliferation	15	5	0.333	1.56 × 10^−3^	0.0260
A	196	141	Apical protein localization	16	3	0.188	2.19 × 10^−3^	0.0313
A	196	141	Nucleotide‐sugar metabolic process	15	3	0.200	2.66 × 10^−3^	0.0333
A	196	141	Oocyte fate determination	30	3	0.100	3.96 × 10^−3^	0.0362
A	196	141	Apoptotic signaling pathway	39	3	0.077	3.97 × 10^−3^	0.0362
A	196	141	Hippo signaling	49	3	0.061	3.98 × 10^−3^	0.0362
C	77	56	Imaginal disc‐derived leg joint morphogenesis	13	5	0.385	7.6 × 10^−9^	7.6 × 10^−7^
C	77	56	Embryonic hindgut morphogenesis	44	5	0.114	5.6 × 10^−6^	2.8 × 10^−4^
M	184	146	Unsaturated fatty acid biosynthetic process	10	6	0.600	4.5 × 10^−9^	4.5 × 10^−7^
O	241	175	Detection of chemical stimulus involved in sensory perception	151	21	0.139	2.2 × 10^−12^	2.2 × 10^−10^
O	241	175	Terpenoid biosynthetic process	10	7	0.700	1.5 × 10^−10^	7.5 × 10^−9^
O	241	175	Unsaturated fatty acid biosynthetic process	10	6	0.600	1.3 × 10^−8^	4.3 × 10^−7^
O	241	175	Phospholipid biosynthetic process	94	8	0.085	2.5 × 10^−7^	6.3 × 10^−6^
O	241	175	Germ cell migration	41	8	0.195	1.4 × 10^−6^	2.8 × 10^−5^
O	241	175	Signal transduction	1393	35	0.025	2.5 × 10^−4^	4.2 × 10^−3^

*Note:* Candidate genes were identified from genomic regions meeting the 2‐SD regional SNP/ancestry criterion and subsequently intersected with the *Apis mellifera* Amel_HAv3.1 gene annotation using BEDTools. “Candidate genes” denotes the total number of non‐redundant genes overlapping ancestry‐associated regions, whereas “GO‐matched genes” denotes candidate genes represented in the curated honey bee GO annotation database and included in the enrichment analysis. “Annotated” indicates the number of genes in the GO background annotated to the corresponding term, “Significant” indicates the number of candidate genes associated with that term, and “Gene ratio” represents Significant/Annotated. GO enrichment was tested for the Biological Process ontology using *topGO* with the weight01 algorithm and Fisher's exact test; GO terms with fewer than 10 annotated genes were excluded (nodeSize = 10). *p* values are nominal Fisher's exact‐test probabilities, and FDR values were calculated using the Benjamini–Hochberg correction. All terms shown for Ancestries A, C, M, and O passed the FDR < 0.05 threshold.

### 3.5 Spatial Structure, Admixture, and Isolation by Distance in U.S. Honey

To evaluate how genomic diversity and spatial structure shape population‐wide patterns in U.S. honey bees, we examined the relationships between heterozygosity and admixture, as well as between genetic relatedness and geographic distance (Figure 5). Across all samples, heterozygosity was strongly associated with admixture proportion (linear regression: β = 3.09 ± 0.04 SE, *R* ^2^ = 0.78, *F*_1,2092_ = 7499, *p* < 2 × 10^−16^), indicating a robust positive relationship between genomic diversity and admixture (Figure 3A). When stratified by major lineage, this relationship differed markedly. Individuals assigned to lineage C exhibited a strong positive association between heterozygosity and admixture (β = 3.02 ± 0.06 SE, *p* < 2 × 10^−16^), whereas lineage A showed a weaker but significant negative relationship (β = −0.95 ± 0.27 SE, *p* = 9.0 × 10^−4^), highlighting lineage‐specific differences in how heterozygosity relates to admixture patterns.

**FIGURE 5:**
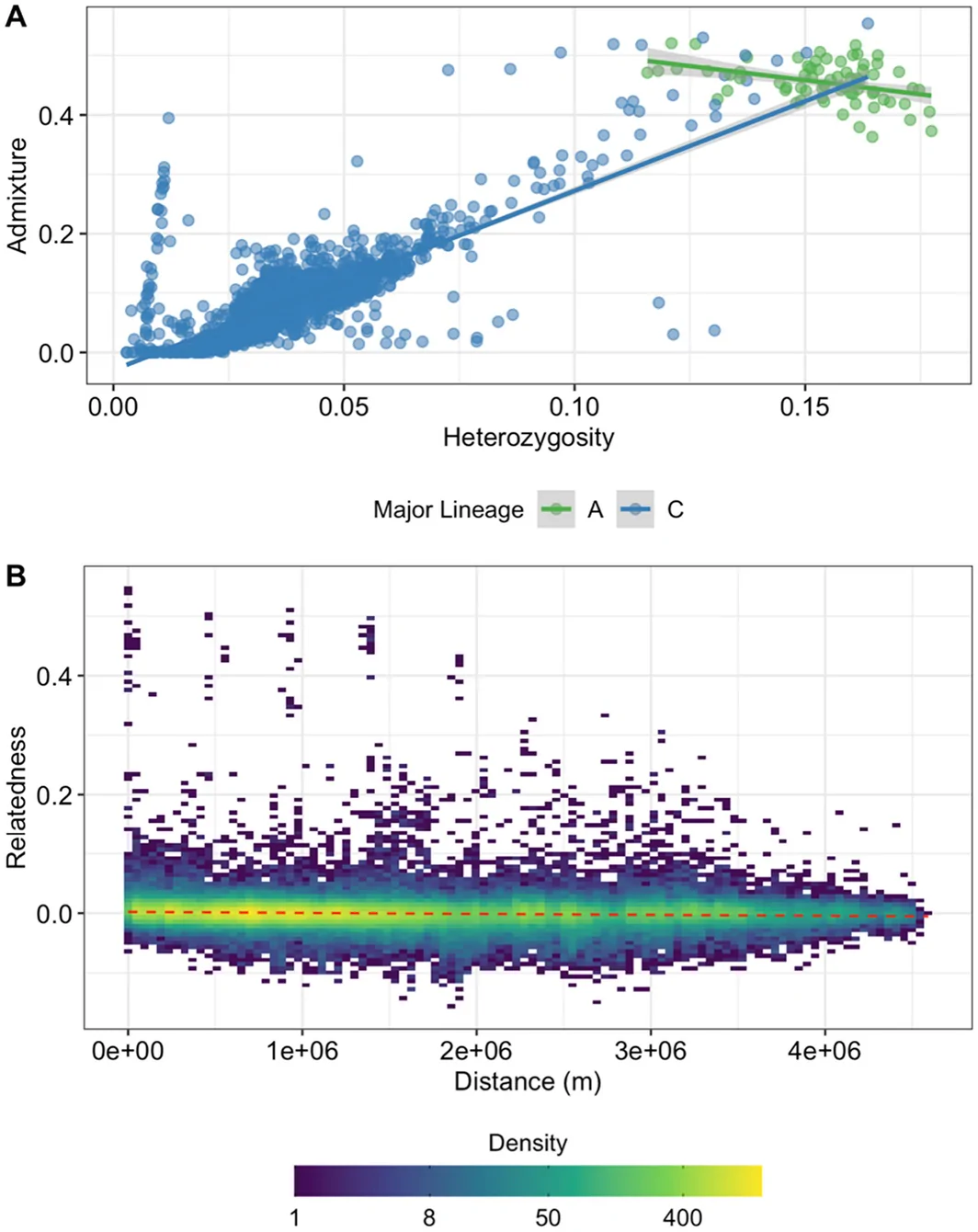
Genomic diversity and spatial genetic structure in a diverse honey bee population. (A) Relationship between individual heterozygosity and ancestry proportion (admixture) for the two major honey bee lineages. Samples are plotted separately for A‐ and C‐lineage ancestry because ancestry proportions are complementary (i.e., an increase in A‐lineage ancestry corresponds to a decrease in C‐lineage ancestry), allowing the association between heterozygosity and admixture to be visualized for each lineage independently. Solid lines represent linear regression fits with 95% confidence intervals. (B) Relationship between pairwise genetic relatedness and geographic distance among all sampled individuals. Color intensity indicates the density of pairwise comparisons, and the dashed red line denotes zero relatedness.

We also detected a significant but weak pattern of isolation by distance across the diverse honey bee population (Figure 5B and Figure S6). Pairwise genetic relatedness declined with increasing geographic distance (linear regression: β = −1.61 × 10^−9^ ± 4.99 × 10^−11^ SE, *F*_1,169,651_ = 1042, *p* < 2.2 × 10^−16^), indicating reduced genetic similarity among individuals separated by larger distances. Although the effect size was small, geographic distance explained a measurable proportion of the variance in relatedness (*R* ^2^ = 0.006), consistent with widespread gene flow and admixture that weaken spatial genetic structure while preserving a detectable isolation‐by‐distance signal.

### 3.6 Directional Gene Flow and Migration Dynamics

TreeMix analysis was used to evaluate covariance patterns and potential migration among the five geographic/regional groups. The working model allowed up to 20 migration events, and the inferred migration relationships were projected onto the U.S. states represented within each cluster (Figure 6 and Figure S2). This framework was used to test whether lineage expansion occurred through directional gene flow and to identify populations acting as major sources or sinks of ancestry (Figure 6A,B). Regional covariance analysis revealed pronounced heterogeneity in shared genetic drift across the United States (Table S1). The strongest between‐region covariance was observed between southern and southwestern populations (mean covariance = 2.53 × 10^−3^; total covariance = 0.0152; *n* = 6 links), exceeding all other regional connections by more than an order of magnitude. Elevated within‐region covariance was also detected in the South (total covariance = 0.00309), followed by the Southwest (0.00181) and West (0.00050), indicating strong regional coherence, whereas covariance involving eastern and midwestern regions was consistently weaker (Table S1). TreeMix analysis revealed substantial genetic connectivity among U.S. honey bee regions, with multiple migration edges connecting branches across geographic regions (Figure S8). The inferred tree showed greater genetic drift for several southwestern populations, including New Mexico, whereas many northeastern and Midwestern states occupied branches with lower drift. Migration edges indicated gene flow across geographically separated regions, consistent with extensive population connectivity and movement among U.S. honey bee populations.

**FIGURE 6:**
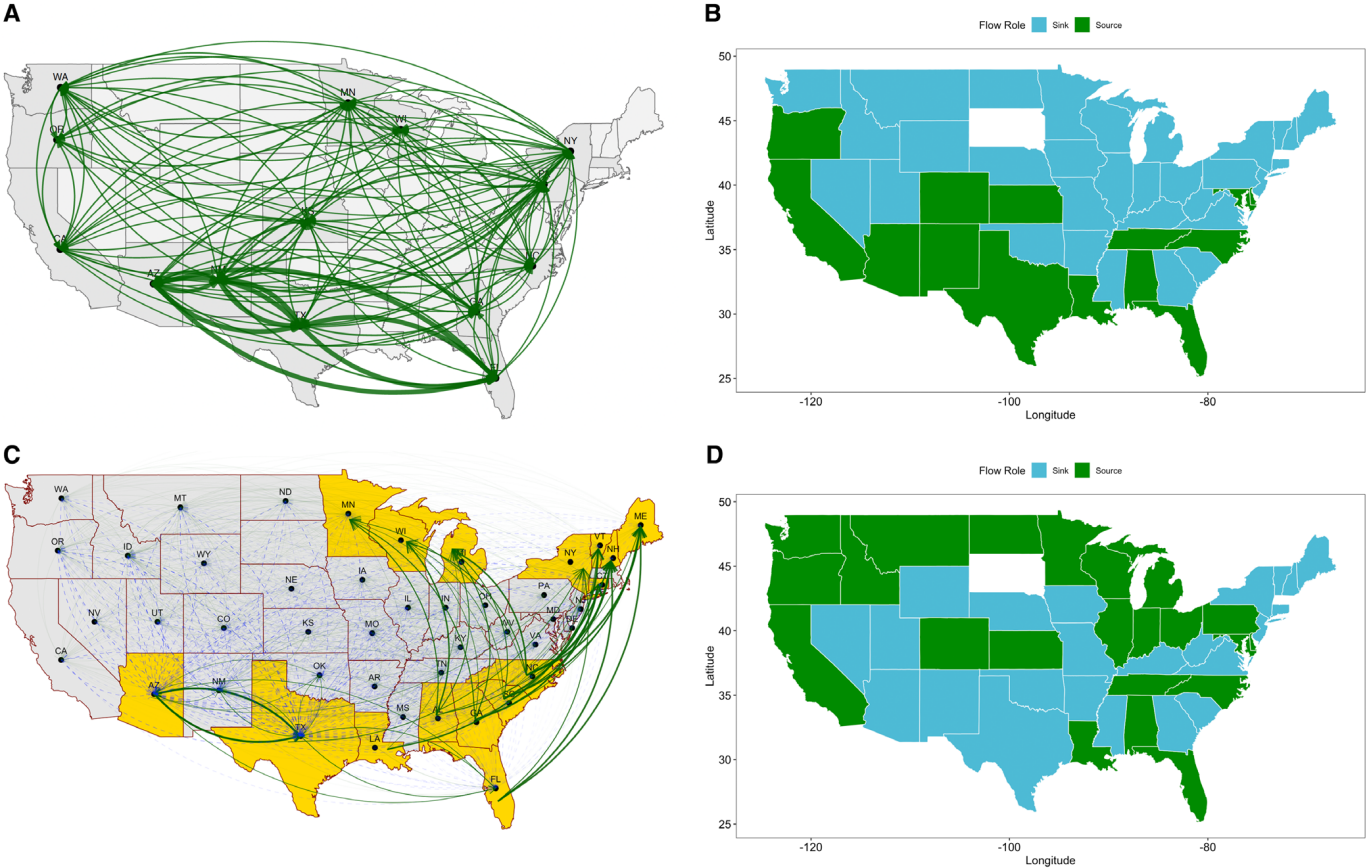
Geographic representation of TreeMix covariance patterns among U.S. honey bee populations. Panels (A, B) show the inferred source and sink roles of U.S. states based on TreeMix covariance patterns for the C‐lineage analysis. Panels (C, D) show the geographic representation of inferred TreeMix migration edges for the A‐lineage analysis. TreeMix was fitted using five geographic clusters with up to 20 migration events, and the inferred cluster‐level relationships were projected onto the states represented within each cluster for geographic visualization. Arrows indicate the direction of inferred migration edges, while colors distinguish source and sink roles.

For the C‐lineage, inferred migration edges did not reveal a consistent unidirectional regional pattern. Instead, gene flow was bidirectional, with prominent connections from eastern and western regions into southern populations. This diffuse structure likely reflects the long history of C‐lineage use in U.S. breeding programs, its dominance in commercial queen exchange, and its widespread temporal establishment across managed populations. In contrast, A‐lineage migration patterns exhibited clearer directional structure, with multiple southern populations contributing ancestry to northern populations. Residual covariance analyses indicated that population relationships could not be fully explained by a strictly bifurcating tree, consistent with admixture and migration. The working TreeMix model allowed up to 20 migration events, and the inferred migration relationships showed a pronounced south‐to‐north pattern, with southern states such as Texas (TX), Arizona (AZ), and Florida (FL) contributing ancestry to northern populations including Michigan (MI), New York (NY), and Vermont (VT). Consistent with this pattern, covariance estimates were highest for TX (mean covariance = 0.047), AZ (0.031), and FL (0.027), underscoring their central role in the dissemination of A‐lineage ancestry.

Integrating ancestry inference from RFMix with migration edges inferred by TreeMix revealed concordant genome‐wide patterns of admixture shaping the genetic structure of U.S. honey bee populations (Figure 6C,D and Figure S8). RFMix profiles showed that A‐lineage ancestry was most prevalent in southern populations and increased gradually toward northern regions, whereas C‐lineage ancestry predominated in northern populations and extended southward into temperate regions. TreeMix‐inferred migration edges were consistent with these ancestry gradients, providing independent support for ongoing directional gene flow between regions. Together, these analyses demonstrate that U.S. honey bee populations are shaped by both regional coherence and long‐distance migration, with southern populations particularly in the South and Southwest playing a dominant role in structuring genome‐wide ancestry patterns.

### 3.7 Association Between Climate and Lineage Introgression

Annual minimum temperature was significantly associated with both the extent and persistence of lineage introgression (Table 2; Figure 7). Global A‐lineage ancestry proportion increased by approximately 0.012 for each 1°C increase in annual minimum temperature (*β* = 0.0117 ± 0.0006, *R* ^2^ = 0.142, *p* < 0.001), indicating that warmer regions retained a greater proportion of A‐lineage ancestry. Likewise, mean A‐lineage haplotype length increased by approximately 1.28 Mb per 1°C increase in annual minimum temperature (*β* = 1.283 ± 0.107, *R* ^2^ = 0.089, *p* < 0.001), indicating that warmer regions also retained longer A‐lineage ancestry tracts.

**TABLE 2:** Linear regression analyses evaluating the association between annual minimum temperature and global ancestry proportion and mean ancestry‐specific haplotype length inferred from RFMix across the four major honey bee evolutionary lineages (A, C, M, and O).

Lineage	Variable	β (Slope)	SE	*t*	*R* ^2^	*p*
Global ancestry proportion						
A	Global	+0.0117	0.0006	19.39	0.142	< 0.001
C	Global	−0.0129	0.0007	−19.40	0.142	< 0.001
M	Global	+0.0016	0.0002	10.70	0.048	< 0.001
O	Global	−0.0004	0.0001	−4.29	0.008	< 0.001
Mean ancestry‐specific haplotype length (Mb)						
A	MHL	+1.283	0.107	12.04	0.089	< 0.001
C	MHL	−3.600	0.173	−20.75	0.159	< 0.001
M	MHL	+0.284	0.049	5.85	0.015	< 0.001
O	MHL	−0.074	0.045	−1.64	0.001	0.102

*Note:* β represents the estimated change in the response variable for each 1°C increase in annual minimum temperature. SE = standard error of the regression coefficient; *t* = *t‐*statistic; *R* ^2^ = coefficient of determination; *p* = significance level of the regression. Positive β values indicate increasing ancestry proportion or haplotype length with increasing temperature, whereas negative *β* values indicate decreasing ancestry proportion or haplotype length.

Abbreviations: Global, Global ancestry proportion; MHL, Mean haplotype length (Mb).

**FIGURE 7:**
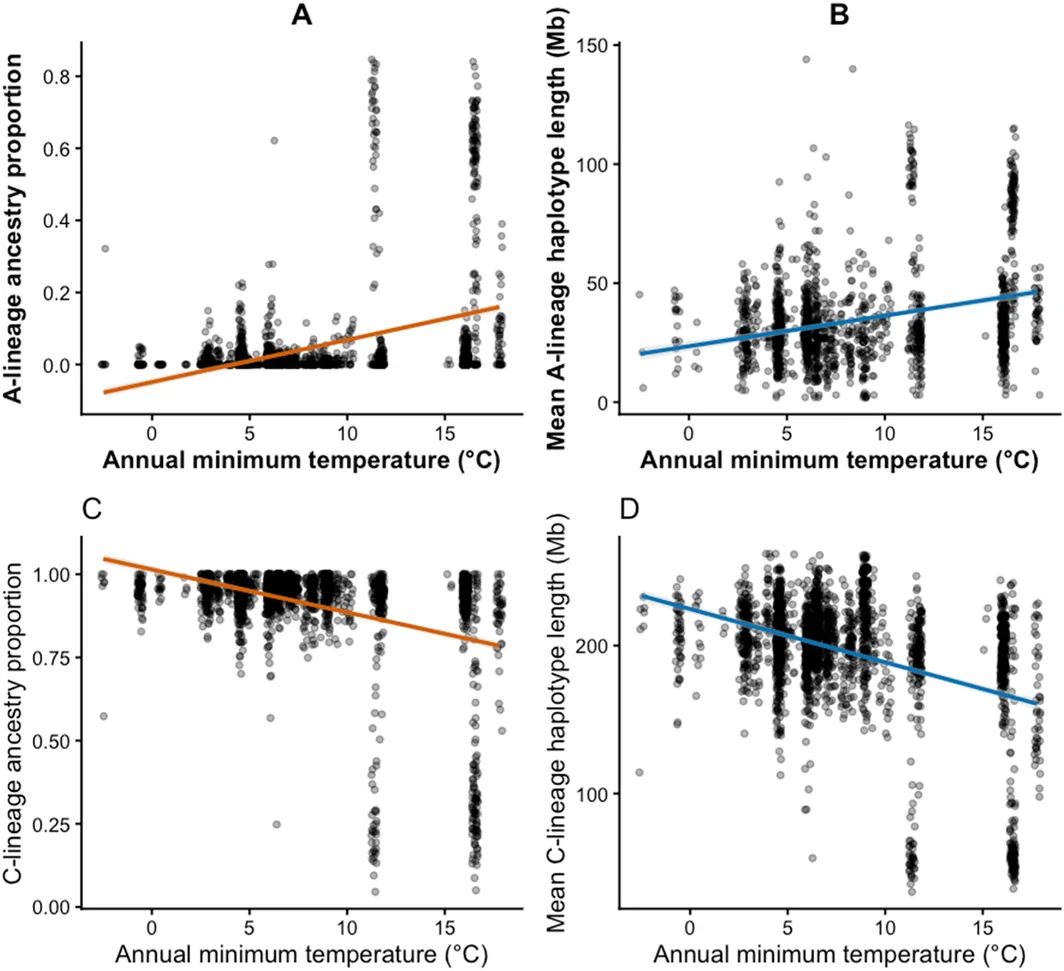
Relationship between annual minimum temperature and A‐ and C‐lineage ancestry in US honey bee populations. (A) Global A‐lineage ancestry proportion estimated by RFMix increased with increasing annual minimum temperature. (B) Mean A‐lineage haplotype length increased with annual minimum temperature, indicating greater retention of longer A‐lineage ancestry tracts in warmer regions. (C) Global C‐lineage ancestry proportion decreased with increasing annual minimum temperature. (D) Mean C‐lineage haplotype length decreased with annual minimum temperature. Together, these patterns indicate opposing relationships between annual minimum temperature and A‐ versus C‐lineage ancestry proportions and haplotype lengths.

In contrast, C‐lineage ancestry exhibited the opposite relationship with temperature. Global C‐lineage ancestry proportion decreased by approximately 0.013 for each 1°C increase in annual minimum temperature (*β* = −0.0129 ± 0.0007, *R* ^2^ = 0.142, *p* < 0.001), while mean C‐lineage haplotype length decreased by approximately 3.60 Mb per 1°C increase (*β* = −3.60 ± 0.17, *R* ^2^ = 0.159, *p* < 0.001), indicating that cooler regions retained both higher proportions of C‐lineage ancestry and longer C‐lineage ancestry tracts. Overall, these analyses demonstrate reciprocal associations between annual minimum temperature and the genomic distribution of A‐ and C‐lineage ancestry across U.S. honey bee populations. Because latitude and annual minimum temperature were highly correlated (*r* = −0.92), the independent contributions of climate and geography could not be fully disentangled.

## 4 Discussion

This study provides a comprehensive genomic assessment of A‐lineage ancestry spread across U.S. honey bee populations by integrating haplotype‐level local ancestry inference with directional migration modeling. Using RFMix and TreeMix, we mapped fine‐scale ancestry tracts and revealed strong northward‐directed gene flow from southern source populations, particularly Texas, Arizona, and Florida, toward northern regions such as Michigan and New York. These results demonstrate that A‐lineage ancestry has been widely incorporated into diverse U.S. populations, likely through both natural dispersal and managed colony movement. However, the extent of A‐lineage introgression varied considerably among individuals and populations, ranging from minimal to substantial ancestry contributions. In addition, low levels of inferred A‐lineage ancestry should be interpreted with caution, as some ancestry tracts may reflect ancestral genomic variation shared between the A‐ and C‐lineages rather than recent introgression (Dogantzis et al. 2021).

We further found that annual minimum temperature was positively associated with both global A‐lineage ancestry proportion and mean A‐lineage haplotype length, whereas C‐lineage ancestry exhibited the opposite pattern. Warmer regions retained higher proportions of A‐lineage ancestry and longer, less fragmented A‐lineage ancestry tracts, while cooler regions retained higher proportions of C‐lineage ancestry and longer C‐lineage ancestry tracts. These reciprocal patterns are consistent with climatic filtering influencing the persistence of lineage‐specific introgression across the United States. However, because latitude and minimum temperature were highly correlated, the independent contributions of climate and geography could not be fully disentangled. In addition, continued anthropogenic movement of managed honey bee colonies likely contributes to the persistence and redistribution of A‐lineage ancestry beyond its expected climatic range.

The identification of genomic regions showing enriched A‐lineage ancestry and high covariance migration edges highlights the dynamic nature of introgression and adaptation in honey bees. Collectively, the findings provide valuable insights into the evolutionary forces shaping population structure and offer practical implications for breeding, conservation, and management strategies aimed at sustaining genetically diverse and resilient honey bee stocks across the United States.

### 4.1 Ancestry‐Informed Haplotypes Reveal Genomic Patterns of Adaptation and Provide Resources for Future Trait Mapping

Our haplotype‐level local ancestry analysis revealed widespread integration of A‐lineage genomic segments across U.S. honey bee populations, with elevated A‐lineage ancestry concentrated in southern regions characterized by warmer climates. Several genomic regions consistently retained high A‐lineage ancestry across geographically distinct populations, suggesting that these ancestry tracts may contribute to adaptation to local environmental conditions. These findings are consistent with previous studies showing that African‐derived ancestry can influence traits associated with thermotolerance, disease resistance, and environmental resilience (Avalos et al. 2025; Harpur et al. 2014). The observed geographic distribution of ancestry also supports the role of climate and natural selection in shaping the genomic landscape of managed honey bee populations. The diffuse, bidirectional connectivity observed among C‐lineage clusters is consistent with the widespread use of C‐lineage bees in commercial queen exchange and colony movement, which can facilitate gene flow among geographically distant populations. Accordingly, we interpret the geographic clusters primarily as a framework for comparing patterns of ancestry and connectivity rather than as discrete or reproductively isolated breeding populations. Information on specific queen‐exchange networks, colony movements, and breeding histories is not systematically recorded across the sampled populations and therefore could not be directly incorporated into our analysis.

Fine‐scale local ancestry inference provides a framework for identifying lineage‐specific haplotypes that may underlie adaptive traits (Avalos et al. 2020; Wragg et al. 2022). By leveraging phased genotypes and reference ancestries, our approach identifies genomic regions that have been preferentially retained following admixture and highlights candidate loci for future functional validation. These ancestry‐informed haplotypes complement conventional population genetic analyses by revealing localized introgression signals that are often masked in genome‐wide summaries. Our results are broadly consistent with Nelson et al. (2017), who analyzed Africanized honey bees from Brazil and reported substantial genomic variation in ancestry proportions, including localized regions of elevated European ancestry. Similarly, we identified genomic regions with elevated representation of specific ancestry components across North American populations, providing complementary evidence of heterogeneous ancestry following admixture (Figure 3). Our results also provide a valuable genomic resource for future genome‐wide association studies (GWAS) and genomic prediction analyses. Haplotype‐based markers capture linkage disequilibrium and historical recombination more effectively than individual SNPs, improving the ability to detect lineage‐specific quantitative trait loci and increasing prediction accuracy (Abed and Belzile 2019; Akey et al. 2001; Araujo et al. 2021; Bu et al. 2024; Saelao et al. 2020; Wallberg et al. 2017; Wragg et al. 2022; Yang and Lawson 2025). Although these findings have potential applications in breeding and conservation, our study primarily establishes a genome‐wide framework for characterizing ancestry patterns and identifying candidate adaptive haplotypes that can be evaluated in future trait‐mapping and functional studies.

## Author Contributions

**Mulusew Fikere:** conceptualization (equal), data curation (equal), formal analysis (equal), methodology (equal), software (equal), validation (equal), visualization (equal), writing – original draft (equal), writing – review and editing (equal). **Dylan K. Ryals:** data curation (supporting), formal analysis (supporting), visualization (supporting). **Andrew H. Paterson:** writing – review and editing (equal). **Brock A. Harpur:** conceptualization (equal), project administration (equal), resources (equal), supervision (equal), writing – review and editing (equal).

## Funding

This study was funded by USDA AFRI (2023‐67013‐38997), FFAR New Innovator, and PDA and North American Pollinator Protection Campaign (NAPPC) grant 40005843.

## Conflicts of Interest

The authors declare no conflicts of interest.

## Supporting information


**Figure S1A:** Genomic data processing, imputation, and SNP quality‐control workflow.
**Figure S1B:** Geographic distribution of sampled honey bee colonies across U.S. states categorized by management status (feral and managed). Bars represent the number of worker honey bee samples collected per state from managed apiaries and feral populations.
**Figure S2:** Haplotype sharing analysis among U.S. states using ancestry data from RFMix. States were colored by the number of shared haplotypes, and green curves depict strong sharing links between states, highlighting regional connectivity and potential routes of genetic exchange.
**Figure S3:** Ancestral proportions after excluding samples outside the 27°–37° latitude range. Individuals with A‐ and C‐lineage ancestry within this region form distinct ancestry proportion peaks, consistent with patterns shown in Figure 2.
**Figure S4:** Hierarchical clustering of honey bee samples based on geographic distance with region, 10 km cluster, and observed heterozygosity (ho) annotations.
**Figure S5:** Pairwise cluster differentiation before and after controlling for ancestry composition.
**Figure S6:** Relationship between mean heterozygosity and latitude after excluding populations with A‐lineage ancestry to determine whether the latitudinal decline in heterozygosity is independent of A‐lineage introgression. Mean expected heterozygosity was calculated for each sampling population and plotted against the population's mean latitude after excluding populations with detectable A‐lineage ancestry (A‐lineage ancestry proportion < 0.01). The solid line represents the fitted linear regression, and the shaded area indicates the 95% confidence interval. Linear regression showed no significant association between mean heterozygosity and latitude (β = −0.000136, *p* = 0.342, *R* ^2^ = 0.004), indicating that the latitudinal decline in heterozygosity observed in the full dataset is largely attributable to populations with A‐lineage ancestry rather than an independent effect of latitude.
**Figure S7:** Functional characterization of genes within Ancestry C|M|O| associated genomic regions. Dot plot showing the top‐ranked Gene Ontology (GO) Biological Process terms identified using topGO. Dot size represents the number of candidate genes associated with each GO term, color indicates nominal significance (−log_10_ *p*), and the *x*‐axis represents the gene ratio. Terms are ranked by nominal Fisher's exact test *p*‐value.
**Figure S8:** Maximum‐likelihood TreeMix analysis of genetic relationships and inferred migration among U.S. honey bee populations. Migration edges indicate inferred gene flow between populations, with edge color representing migration weight, while branch lengths represent genetic drift. The residual covariance plot summarizes the correspondence between observed and model‐predicted covariance, providing an assessment of model fit.
**Figure S9:** Principal component analysis of genome‐wide genetic variation by mitochondrial ancestry among U.S. honey bee samples.

**Table S1:** Mean covariance, total covariance, and number of covariance links among U.S. honey bee regions estimated using TreeMix.

**Table S2:** Gene Ontology (GO) Biological Process enrichment results for genes overlapping ancestry‐associated genomic regions.

## Data Availability

The datasets generated and/or analyzed during the current study are available in the European Variation Archive (EVA) repository under project number PRJEB123676. The associated analysis accession is ERZ2980791. The data are archived and will be publicly available from 31 December 2027. All analysis scripts and code used in this study are available on GitHub at: https://github.com/DrMulusewFikere/Local‐Ancestry‐Inference‐LAI‐.
