# Supplementary material for: Nationwide Tracking of A‐Lineage Spread in Western Honey Bees Using Haplotype Sharing, Local Ancestry Mapping, and Migration Inference
Source: Ecol Evol. 2026 Oct 30;16(11):e74574. doi: 10.1002/ece3.74574 (PMC13722957; doi:10.1002/ece3.74574)
Supplement: Supplementary file 1 [file ECE3-16-e74574-s002.docx]

**Supplementary Figures**

Figure S1A. Genomic data processing, imputation, and SNP quality-control workflow.

### Figure S1B. Geographic distribution of sampled honey bee colonies across U.S. states categorized by management status (feral and managed). Bars represent the number of worker honey bee samples collected per state from managed apiaries and feral populations.

Figure S2. Haplotype sharing analysis among U.S. states using ancestry data from RFMix. States were colored by the number of shared haplotypes, and green curves depict strong sharing links between states, highlighting regional connectivity and potential routes of genetic exchange.

Figure S3. Ancestral proportions after excluding samples outside the 27-37^o^ latitude range. Individuals with A- and C-lineage ancestry within this region form distinct ancestry proportion peaks, consistent with patterns shown in Figure 2.

Figure S4. Hierarchical clustering of honey bee samples based on geographic distance with region, 10 km cluster, and observed heterozygosity (ho) annotations

Figure S5. Pairwise cluster differentiation before and after controlling for ancestry composition.

Mean genome-wide pairwise FST among five regional honey bee clusters across the United States. Fst was calculated for each cluster pair using genome-wide SNP data with the Weir and Cockerham estimator implemented in PLINK. Analyses were performed using (i) all samples within each cluster pair (ALL) and (ii) subsets restricted to individuals assigned to A-lineage or C-lineage ancestry based on ancestry assignments. For each comparison, the mean Fst across SNPs is shown.

**Figure S6. Relationship between mean heterozygosity and latitude after excluding populations with A-lineage ancestry to determine whether the latitudinal decline in heterozygosity is independent of A-lineage introgression. Mean expected heterozygosity was calculated for each sampling population and plotted against the population's mean latitude after excluding populations with detectable A-lineage ancestry (A-lineage ancestry proportion < 0.01). The solid line represents the fitted linear regression, and the shaded area indicates the 95% confidence interval. Linear regression showed no significant association between mean heterozygosity and latitude (**$\beta$ **= -0.000136, P = 0.342, R² = 0.004), indicating that the latitudinal decline in heterozygosity observed in the full dataset is largely attributable to populations with A-lineage ancestry rather than an independent effect of latitude.**

**Figure S7. Functional characterization of genes within Ancestry C | M | O | associated genomic regions. Dot plot showing the top-ranked Gene Ontology (GO) Biological Process terms identified using topGO. Dot size represents the number of candidate genes associated with each GO term, color indicates nominal significance (-log₁₀ P), and the x-axis represents the gene ratio. Terms are ranked by nominal Fisher’s exact test P-value.**

Figure S8. Maximum-likelihood TreeMix analysis of genetic relationships and inferred migration among U.S. honey bee populations. Migration edges indicate inferred gene flow between populations, with edge color representing migration weight, while branch lengths represent genetic drift. The residual covariance plot summarizes the correspondence between observed and model-predicted covariance, providing an assessment of model fit.

Figure S9. Principal component analysis of genome-wide genetic variation by mitochondrial ancestry among U.S. honey bee samples.
