# Supplementary material for: Nationwide Tracking of A‐Lineage Spread in Western Honey Bees Using Haplotype Sharing, Local Ancestry Mapping, and Migration Inference
Source: Ecol Evol. 2026 Oct 30;16(11):e74574. doi: 10.1002/ece3.74574 (PMC13722957; doi:10.1002/ece3.74574)
Supplement: Supplementary file 2 [file ECE3-16-e74574-s003.docx]

### **Supplementary Tables**

Table S1. Mean covariance, total covariance, and number of covariance links among U.S. honey bee regions estimated using TreeMix.

| RegionA | RegionB | mean_cov | total_cov | nLinks |
| --- | --- | --- | --- | --- |
| South | Southwest | 0.00253 | 0.0152 | 6 |
| South | South | 0.000771 | 0.00309 | 4 |
| Southwest | Southwest | 0.00181 | 0.00181 | 1 |
| Midwest | Southwest | 0.000407 | 0.000814 | 2 |
| North | West | 0.0000863 | 0.000777 | 9 |
| East | Southwest | 0.000315 | 0.000629 | 2 |
| West | West | 0.000167 | 0.000502 | 3 |
| East | South | 0.0000851 | 0.000341 | 4 |
| South | West | 0.0000599 | 0.000299 | 5 |
| North | Southwest | 0.0000992 | 0.000298 | 3 |
| North | South | 0.0000321 | 0.000257 | 8 |
| Midwest | South | 0.0000842 | 0.000253 | 3 |
| Southwest | West | 0.000101 | 0.000101 | 1 |
| North | North | 0.0000295 | 0.0000886 | 3 |
| East | Midwest | 0.0000686 | 0.0000686 | 1 |
| Midwest | North | 0.0000281 | 0.0000562 | 2 |
| Midwest | West | 0.0000281 | 0.0000562 | 2 |
| East | West | 0.0000164 | 0.0000328 | 2 |
| East | North | 0.00000981 | 0.0000294 | 3 |
